# The quality of systematic reviews about interventions for refractive error can be improved: a review of systematic reviews

**DOI:** 10.1186/s12886-017-0561-9

**Published:** 2017-09-05

**Authors:** Evan Mayo-Wilson, Sueko Matsumura Ng, Roy S. Chuck, Tianjing Li

**Affiliations:** 1 Department of Epidemiology, Johns Hopkins University Bloomberg School of Public Health, 615 North Wolfe Street, Baltimore, MD 21205 USA; 2 Department of Ophthalmology and Visual Sciences, Albert Einstein College of Medicine, Montefiore Medical Center, 3332 Rochambeau Avenue, Centennial, Room 306, New York, NY 10467 USA

**Keywords:** Systematic review standards, Refractive error, Clinical guidelines, Research waste

## Abstract

**Background:**

Systematic reviews should inform American Academy of Ophthalmology (AAO) Preferred Practice Pattern® (PPP) guidelines. The quality of systematic reviews related to the forthcoming Preferred Practice Pattern® guideline (PPP) *Refractive Errors & Refractive Surgery* is unknown. We sought to identify reliable systematic reviews to assist the AAO *Refractive Errors & Refractive Surgery* PPP.

**Methods:**

Systematic reviews were eligible if they evaluated the effectiveness or safety of interventions included in the 2012 PPP *Refractive Errors & Refractive Surgery*. To identify potentially eligible systematic reviews, we searched the Cochrane Eyes and Vision United States Satellite database of systematic reviews. Two authors identified eligible reviews and abstracted information about the characteristics and quality of the reviews independently using the Systematic Review Data Repository. We classified systematic reviews as “reliable” when they (1) defined criteria for the selection of studies, (2) conducted comprehensive literature searches for eligible studies, (3) assessed the methodological quality (risk of bias) of the included studies, (4) used appropriate methods for meta-analyses (which we assessed only when meta-analyses were reported), (5) presented conclusions that were supported by the evidence provided in the review.

**Results:**

We identified 124 systematic reviews related to refractive error; 39 met our eligibility criteria, of which we classified 11 to be reliable. Systematic reviews classified as unreliable did not define the criteria for selecting studies (5; 13%), did not assess methodological rigor (10; 26%), did not conduct comprehensive searches (17; 44%), or used inappropriate quantitative methods (3; 8%). The 11 reliable reviews were published between 2002 and 2016. They included 0 to 23 studies (median = 9) and analyzed 0 to 4696 participants (median = 666). Seven reliable reviews (64%) assessed surgical interventions.

**Conclusions:**

Most systematic reviews of interventions for refractive error are low methodological quality. Following widely accepted guidance, such as Cochrane or Institute of Medicine standards for conducting systematic reviews, would contribute to improved patient care and inform future research.

**Electronic supplementary material:**

The online version of this article (10.1186/s12886-017-0561-9) contains supplementary material, which is available to authorized users.

## Background

Systematic reviews of interventions are used to inform clinical guidelines [1], to set research priorities [2], and to help patients and clinicians make healthcare decisions. Well-conducted systematic reviews focus on clear research questions and use reproducible methods to identify, select, describe, and synthesize information from relevant studies [3]. Such reviews can reduce uncertainty about the effectiveness of interventions, lead to faster adoption of safe and effective interventions, and identify research needs. On the other hand, poorly-conducted systematic reviews may be harmful if they contribute to suboptimal patient care or promote unnecessary research.

Each year, the number of published systematic reviews continues to increase throughout medicine, yet the quality of systematic reviews is highly variable [2, 4]. To be considered reliable, systematic reviews must be conducted using methods that minimize bias and error in the review process, and must be reported completely and transparently [3].

Because refractive error is the leading cause of visual impairment globally [5], effective interventions to correct refractive error are important to patients and to optometrists and ophthalmologists. The American Academy of Ophthalmology (AAO) updated their Preferred Practice Pattern (PPP) for *Refractive Errors & Refractive Surgery* in 2017. To assist the AAO in this task, Cochrane Eyes and Vision @ United States (CEV@US) investigators sought to identify reliable systematic reviews about interventions for refractive error using a database of systematic reviews in eyes and vision [6].

## Methods

Systematic reviews were eligible for this project if they evaluated the effectiveness or safety of interventions for refractive error that were included in the 2012 AAO’s PPP *Refractive Errors & Refractive Surgery* [7]. We excluded systematic reviews related to cataract removal with implantation of an intraocular lens for correcting refractive error because this topic is covered in another PPP. We included all reports that claimed to be systematic reviews. Otherwise, we defined a systematic review as “a scientific investigation that focuses on a specific question and uses explicit, pre-specified scientific methods to identify, select, assess, and summarize similar but separate studies [8].” Consistent with this definition, eligible studies were not required to include meta-analyses. Whenever a systematic review had been updated since the initial publication, we reviewed the most recent update.

CEV@US maintains a database of systematic reviews related to vision research and eye care (see Online Additional file 1 for search strategies used to identify reviews). We conducted an initial search of PubMed and Embase in 2007, and we updated the search in 2009, 2012, 2014, and on May 15, 2016 [9]. Two individuals identified systematic reviews related to vision research and eye care. For the 2007, 2009, and 2012 searches, two people identified relevant refractive error systematic reviews independently. For the 2014 and 2016 searches, one person identified potentially eligible refractive error reviews, then a second person verified that the reports were eligible. Differences were resolved through discussion that sometimes included another team member.

We developed a data abstraction form that included components of the Critical Appraisal Skills Programme (CASP) [10], the Assessment of Multiple Systematic Reviews (AMSTAR) [11], and the Preferred Reporting Items for Systematic Reviews and Meta-Analyses (PRISMA) [12]. We adapted a version of the form used in previous studies [4, 13] (see Online Additional file 2 for a copy of the electronic form). In addition to the results presented in this paper, we recorded other descriptive information about the reviews. We entered data electronically using the Systematic Review Data Repository (SRDR) [14].

Two authors (EMW and SN) abstracted data independently from eligible systematic reviews and resolved discrepancies through discussion. We classified systematic reviews as “reliable” when the systematic reviewers had (1) defined criteria for the selection of studies, (2) conducted comprehensive literature searches for eligible studies, (3) assessed the methodological quality (risk of bias) of the included studies, (4) used appropriate methods for meta-analyses (which we assessed only when meta-analyses were reported), and (5) presented conclusions that were supported by the evidence provided in the review. We considered a systematic review “unreliable” when one or more of these criteria were not met.

## Results

**Fig. 1:**
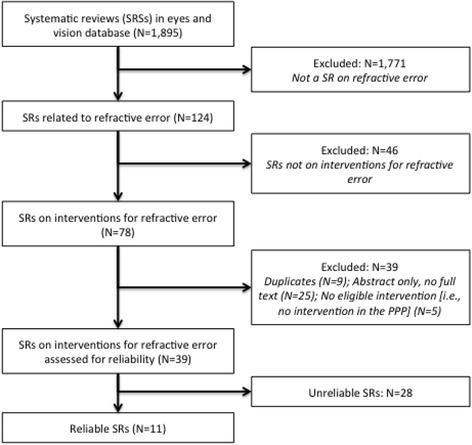
Flow chart showing the identification of systematic reviews of interventions for refractive error

Of 124 systematic reviews in our database and classified as related to refractive error, 39 met our eligibility criteria (Fig. 1). Systematic reviews that met our inclusion criteria were published between 1996 and 2014 (median = 2011). They included between 0 and 309 studies (median = 9.5). In 13/39 (33%) systematic reviews, we could not ascertain how many participants contributed data; the 26 remaining reviews included data from 0 to 9336 participants.

Of the 39 systematic reviews, 22 (56%) assessed surgical interventions, including reviews of LASEK or LASIK (18; 45%) and intraocular lenses (4; 10%). Other reviews assessed orthokeratology (10; 26%), atropine (2; 5%), monovision (1; 3%), contact lenses (1; 3%), spectacles (1; 3%), and multiple interventions (2; 5%). Participants with myopia were included in 30 (77%) reviews. Reviews also included participants with astigmatism (15; 38%), hyperopia (10; 26%), and presbyopia (3; 8%).

**Fig. 2:**
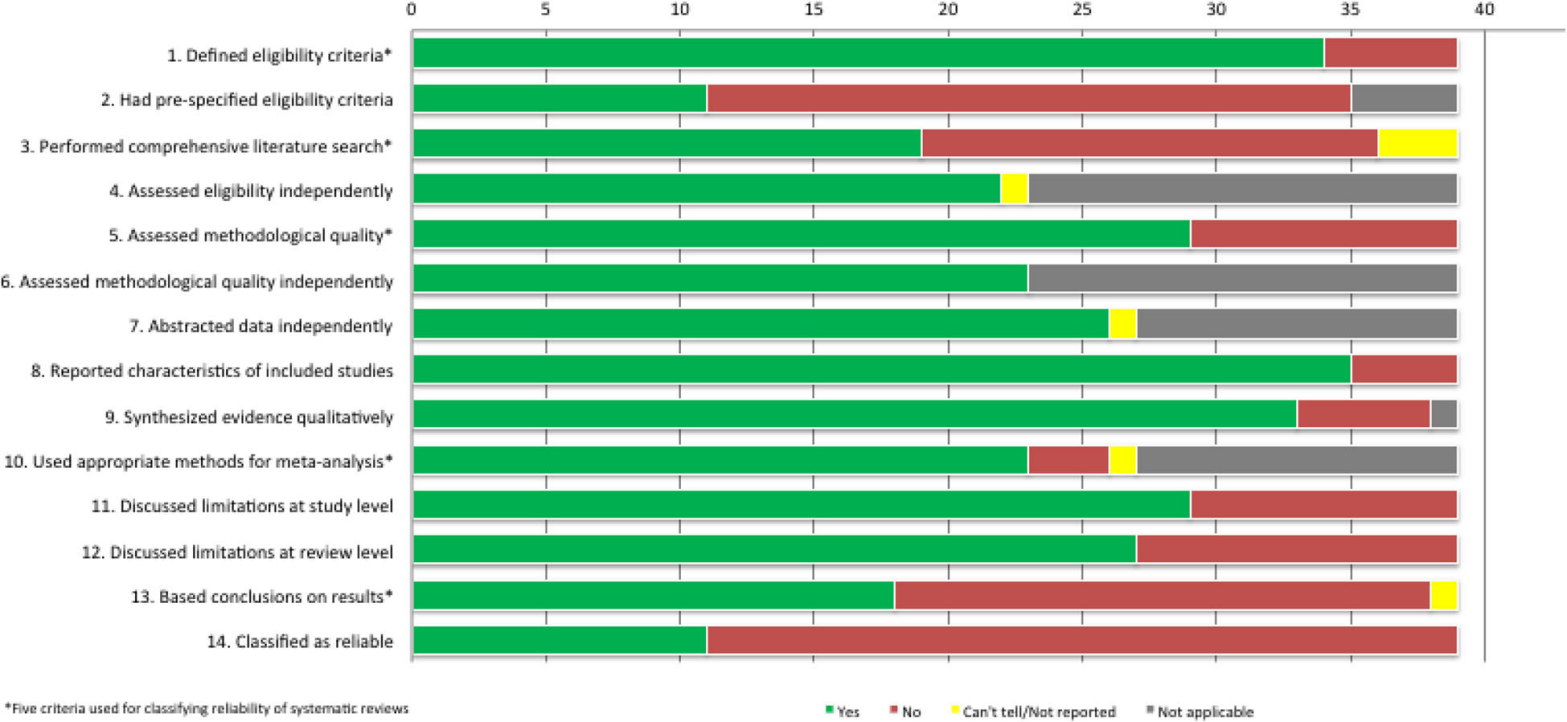
Assessment of reliability criteria for 40 systematic reviews on interventions for refractive error

Of the 39 eligible systematic reviews, 11 (28%) were classified as reliable [15–25], and 28 (72%) were classified as unreliable (Fig. 2) [26–53]. One of the reliable reviews did not include any studies. All 11 reliable systematic reviews assessed interventions for myopia, either to slow progression of myopia in children (5 reviews), to correct myopia surgically (5 reviews), or to treat choroidal neovascularization secondary to pathologic myopia (1 review). The 11 reliable systematic reviews were published between 2002 and 2016, included data from 0 to 23 studies (median = 9), and analyzed data for 0 to 4696 participants (median = 666). Of the 11 systematic reliable reviews, 7 (64%) assessed surgical interventions, and included some of the same studies. Different groups of reviewers reached contradictory conclusions about the safety and effectiveness of LASEK compared with PRK (Table 1).

**Table 1:** Objectives, Participants, Interventions, Outcomes and Conclusions of the Reliable Systematic Reviews on Interventions for Refractive Error and Refractive Surgery (*N* = 11)

Study ID	Objective(s)	Condition(s)	Intervention Comparisons	Outcome	Number of Studies; Participants; Eyes	Conclusion(s) from the abstract
Pharmaceuticals						
Li 2014 [17]	“To conduct a meta-analysis on the effects of atropine in slowing myopia progression and to compare Asian and white children and randomized controlled trials (RCTs) and observational studies.”	Myopia (children)	Atropine compared with placebo or non-atropine treatment	Refractive error	11; 1815; Not reported	“Atropine could significantly slow myopia progression in children, with greater effects in Asian than in white children. Randomized controlled trials and cohort studies provided comparable effects.”
Spectacles						
Li 2011 [22]	“Multifocal lenses (MLs) are advocated as a substitute for single vision lenses (SVLs) to slow myopia progression in children, but results vary greatly across studies.”	Myopia (children)	Multifocal lenses compared with single vision lenses	Visual acuity, axial length	9; 1464; Not reported	“A meta-analysis of nine of these trials showed that MLs with powers ranging from þ1.50 to þ2.00D were associated with a statistically significantly decrease in myopia progression in school-aged children compared with SVLs. The benefit was greater in children with a higher level of myopia at baseline and sustained for a minimum of 24 months. Asian children appeared to have greater benefit from intervention with MLs than white children.”
Surgery						
*Barsam 2014 [15]	“To compare excimer laser refractive surgery and phakic IOLs for the correction of moderate to high myopia by evaluating postoperative uncorrected visual acuity, refractive outcome, potential loss of best spectacle corrected visual acuity (BSCVA) and the incidence of adverse outcomes.”	Myopia	Phakic intraocular lenses compared with excimer laser surgical	Visual acuity (UCVA); Need for correction; Patient satisfaction; Quality of life; Cost	3; 132; 228	“The results of this review suggest that, at one year post surgery, phakic IOLs are safer than excimer laser surgical correction for moderate to high myopia in the range of −6.0 to −20.0 D and phakic IOLs are preferred by patients. While phakic IOLs might be accepted clinical practice for higher levels of myopia (greater than or equal to 7.0 D of myopic spherical equivalent with or without astigmatism), it may be worth considering phakic IOL treatment over excimer laser correction for more moderate levels of myopia (less than or equal to 7.0 D of myopic spherical equivalent with or without astigmatism). Further RCTs adequately powered for subgroup analysis are necessary to further elucidate the ideal range of myopia for phakic IOLs. This data should be considered alongside comparative data addressing long-term safety as it emerges.”
*Li 2016 [16]	“The objective of this review is to compare LASEK versus PRK for correction of myopia by evaluating their efficacy and safety in terms of postoperative uncorrected visual acuity, residual refractive error, and associated complications.”	Myopia; Astigmatism	LASEK compared with PRK	Visual acuity (UCVA, BCVA); Refractive error; Adverse events	11; 428; 866	“Uncertainty surrounds differences in efficacy, accuracy, safety, and adverse effects between LASEK and PRK for eyes with low to moderate myopia. Future trials comparing LASEK versus PRK should follow reporting standards and follow correct analysis. Trial investigators should expand enrollment criteria to include participants with high myopia and should evaluate visual acuity, refraction, epithelial healing time, pain scores, and adverse events.”
*Settas 2012 [19]	“The objectives of this review were to determine whether PRK or LASIK leads to more reliable, stable and safe results when correcting a hyperopic refractive error.”	Hyperopia; Astigmatism	LASIK compared with PRK	Visual acuity (UCVA, BCVA); Refractive error; Adverse events	0; 0; 0	“No robust, reliable conclusions could be reached, but the non-randomised trials reviewed appear to be in agreement that hyperopic-PRK and hyperopic-LASIK are of comparable efficacy. High quality, well-planned open RCTs are needed in order to obtain a robust clinical evidence base.”
Shortt 2006 [23]	“The aim of this review was to compare the effectiveness and safety of PRK and LASIK for correction of myopia.”	Myopia; Astigmatism	LASIK compared with PRK	Visual acuity (UCVA); Refractive error; Quality of life; Adverse events	6; 666; 417	“LASIK gives a faster visual recovery than PRK but the effectiveness of these two procedures is comparable. Further trials using contemporary techniques are required to determine whether LASIK and PRK are equally safe.”
*Shortt 2013 [18]	“To compare the effectiveness and safety of LASIK and PRK for correction of myopia by examining post-treatment uncorrected visual acuity, refractive outcome, loss of best spectacle-corrected visual acuity, pain scores, flap complications in LASIK, subepithelial haze, adverse events, quality of life indices and higher order aberrations.”	Myopia; Astigmatism	LASIK compared with PRK	Visual acuity (UCVA, BCVA); Refractive error; Quality of life; Adverse events	13; 1135; 1923	“LASIK gives a faster visual recovery and is a less painful technique than PRK. The two techniques appear to give similar outcomes one year after surgery. Further trials using contemporary techniques are required to determine whether LASIK and PRK as currently practised are equally safe. Randomising eyes to treatment is an efficient design, but only if analysed properly. In future trials, more efforts could be made to mask the assessment of outcome.”
Virgili 2005 [24]	“The primary objective of this review was to examine the effects of laser photocoagulation for CNV associated with pathologic myopia. A secondary objective was to compare the effects of different photocoagulation techniques.”	Myopia	Laser photocoagulation compared with no treatment or sham treatment	Visual acuity; Quality of life; Functioning; Adverse events	2; 96; 97	“Despite its use over several years the effectiveness of laser photocoagulation for myopic CNV has not been established. Although there was a suggestion of short-term effectiveness in one small study on non-subfoveal CNV the results were potentially biased. Observational studies suggest that the enlargement of the atrophic laser scar after laser treatment of non-subfoveal CNV could be a potentially vision- threatening long-term complication, even in eyes free of CNV recurrence.”
Multiple interventions						
Saw 2002 [25]	“To evaluate the efficacy of interventions such as eyedrops, bifocal lenses, or contact lenses in retarding the progression of myopia in myopic children.”	Myopia (children)	“Interventions to retard the progression of myopia”	Adverse events	10; 1612; Not reported	“The latest evidence from randomized clinical trials does not provide sufficient information to support interventions to prevent the progression of myopia. Long-term large-scale double-masked randomized clinical trials, including cycloplegic refraction, are needed before any recommendations about interventions in clinical practice to prevent high myopia in myopic children are considered.”
*Walline 2011 [20]	“To assess the effects of several types of interventions, including eye drops, undercorrection of nearsightedness, multifocal spectacles and contact lenses, on the progression of nearsightedness in myopic children younger than 18 years. We compared the interventions of interest with each other, to single vision lenses (SVLs) (spectacles), placebo or no treatment.”	Myopia (children)	Bifocal soft contact lenses (BSCLs), rigid gas permeable contact lenses (RGPCLs) and corneal reshaping (orthokeratology) contact lenses; Bifocal lenses (spectacles), progressive addition lenses (PALs) and undercorrection of myopia; Pharmaceutical agents	Refractive error; Quality of life; Adverse events; Cost	23; 4696; Not reported	“The most likely effective treatment to slow myopia progression thus far is anti-muscarinic topical medication. However, side effects of these medications include light sensitivity and near blur. Also, they are not yet commercially available, so their use is limited and not practical. Further information is required for other methods of myopia control, such as the use of corneal reshaping contact lenses or bifocal soft contact lenses (BSCLs) with a distance center are promising, but currently no published randomized clinical trials exist.”
Other interventions						
*Wei 2011 [21]	“To assess the effectiveness and safety of acupuncture in slowing the progression of myopia in children and adolescents.”	Myopia (children and adolescents)	Acupuncture	Refractive error; Adverse events; Cost; Axial length; Corneal radius	2; 131; Not reported	“Two trials are included in this review but no conclusions can be drawn for the benefit of co-acupressure for slowing progress of myopia in children. Further evidence in the form of RCTs are needed before any recommendations can be made for the use of acupuncture treatment in clinical use. These trials should compare acupuncture to placebo and have large sample sizes. Other types of acupuncture (such as auricular acupuncture) should be explored further as well as compliance with treatment for at least six months or longer. Axial length elongation of the eye should be investigated for at least one year. The potential to reduce/eliminate pain from acupuncture experienced by children should also be reviewed.”

*Cochrane review

The 28 systematic reviews that we classified as unreliable were published between 1996 and 2016 (see Online Additional file 3 for the data extracted for all included systematic reviews) [26–53]. Unreliable systematic reviews did not define the criteria for selecting studies (5; 13%), did not assess methodological quality of the individual studies (10; 26%), did not conduct comprehensive literature searches for eligible studies (17; 44%), or used inappropriate quantitative methods, such as combining randomized and non-randomized studies for meta-analysis (3; 8%). All systematic reviews that presented conclusions not supported by the data were also classified as unreliable for another reason (20; 51%).

## Discussion

Our investigation revealed that most systematic reviews about interventions for refractive error published to date have been of low methodological quality; consequently, these reviews may result in inappropriate decisions about clinical care and future research. Some of the shortcomings we identified were related to reporting deficiencies. For example, authors of most reviews did not report criteria for selecting studies. Also, many authors did not report essential characteristics of the included studies, such as sample sizes. Other shortcomings were related to both poor reporting and poor conduct. Our findings are thus consistent with evidence that most systematic reviews published to date have been neither conducted nor reported following best practices [54, 55].

Although visual impairment due to uncorrected refractive error is particularly prevalent in low-income countries [5], most of the systematic reviews included in our study addressed interventions not widely available in those countries. Systematic reviewers may have focused on interventions of interest to decision-makers in high-income countries, and there may be few randomized trials of interventions suitable for use in low-income countries and thus available for inclusion in systematic reviews. Although we classified the systematic review reported by Pearce [29] as unreliable, it is the exception among the 40 systematic reviews for having dealt with an intervention with wide applicability.

A comprehensive and reproducible literature search lays the foundation for a high-quality systematic review. To be transparent and reproducible, systematic reviews should list all information sources searched, the exact search terms used, the operators used to combine terms, and the dates of the searches [56]; however, many systematic reviews of interventions for refractive error did not describe the search methods for identifying eligible studies. Moreover, the number of citations retrieved in many systematic reviews was so small that a knowledgeable reader might suspect the search methods were not sensitive. As a rule of thumb, systematic reviewers can expect to retrieve and review about 200 citations (titles and abstracts) and 5 full-text reports to identify one study eligible for inclusion in a systematic review [57].

Reviewers should describe the methodological quality of included studies using a method such as the Cochrane Risk of Bias Tool to identify potential sources of bias that could influence the credibility of results of individual studies and hence systematic reviews and meta-analysis [58, 59]. Many reviews did not mention having assessed the methodological quality of individual studies, and some reviews specified methods that are known to be unreliable (e.g., the Jadad Scale) [60]. Quality scores are problematic because they are inconsistent and lack validity; the same study may be assessed as excellent using one scoring method but poorly using another scoring method [61].

Finally, authors of seven systematic reviews used methods for data synthesis that were inappropriate. Of particular concern are reviews in which outcomes from randomized controlled trials were combined with outcomes from observational studies in the same meta-analysis. For a research question on the effectiveness of an intervention, randomized controlled trials, by design, have more protection against bias than observational studies. When both types of studies are available, systematic reviewers should justify the reasons for relying on observational data as a substitute for or a complement to data from randomized trials. It is important to understand and to appraise the strengths and weaknesses of observational data when they are used to assess the effectiveness of interventions. When dissimilar studies are combined in meta-analysis, the resulting estimates of intervention effects might be meaningless.

Systematic reviews about interventions for refractive error assessed only a few distinct interventions, a finding consistent with other evidence that systematic reviews are duplicative throughout medicine [62]. After completing our review and sharing the results with the AAO PPP, the panel informed us that one reliable systematic review was not relevant because it examined choroidal neovascularization and laser treatment in high myopia, which is considered a retina topic [24]. Furthermore, some of the reliable reviews included only a few small studies and thus provided inconclusive evidence about the effectiveness and safety of the targeted interventions. Although it is possible for reviews to use pre-specified methods and reach different conclusions (e.g., because of differences in outcomes or inclusion criteria), we find it concerning that some of the reliable reviews reached inconsistent conclusions about the same interventions, even though they included some of the same studies.

We searched for systematic reviews using PubMed and Embase, and we might have missed systematic reviews that are not indexed in these databases. It seems unlikely that systematic reviews in non-indexed journals would be of better quality than the reviews we identified, and their inclusion in our study would have been unlikely to lead us a different conclusion. More high-quality evidence is needed to inform clinical guidelines and to improve patient care for this common problem.

Journal editors could help improve the quality of systematic reviews by adopting four requirements. First, editors should require that authors of systematic reviews publish or provide protocols for their reviews that adhere to current best practices (e.g., the Preferred Reporting Items for Systematic Review and Meta-analysis Protocols (PRISMA-P) statement [63]). Second, editors should require that all reports of systematic reviews include the information in the PRISMA statement [64], which should be documented in a completed PRISMA checklist that accompanies every systematic review manuscript and is confirmed by a peer reviewer or a member of the editorial team. By considering only those manuscripts that adhere to these requirements, editors could ensure that readers have the information needed to assess the methodological rigor of systematic reviews and to decide whether to apply their findings to practice. Third, to reduce the publication of reviews that are essentially redundant (i.e., reviews that include the same studies), editors should require that authors explain how their systematic reviews differ from other reviews of the same interventions or, if the systematic reviews are not different, editors should require that authors reference existing systematic reviews about the same topic. Finally, manuscripts that report systematic reviews should be reviewed by people knowledgeable about both systematic reviews and the clinical specialties that the reviews concern. Eight ophthalmology and optometry journals now have editors for systematic reviews, which may improve the quality of reviews in those journals (http://eyes.cochrane.org/associate-editors-eyes-and-vision-journals).

## Conclusions

In conclusion, systematic reviews about interventions for refractive error could benefit patient care and avoid redundant research if the authors of such reviews followed widely accepted guidance, such as Cochrane or Institute of Medicine standards for conducting systematic reviews [3, 65]. The reliable systematic reviews we identified may be useful in updating the AAO PPP and in future updates of the American Optometric Association’s Evidence-Based Clinical Practice Guidelines.

## Additional files

Additional file 1: Search strategies for identifying eyes and vision systematic reviews. (DOCX 42 kb)

Additional file 2: Data extraction form. (PDF 676 kb)

Additional file 3: Review characteristics. (XLSX 14 kb)

## References

[1] Institute of Medicine (2011). Clinical practice guidelines we can trust.

[2] Li T, Vedula SS, Scherer R, Dickersin K (2012). What comparative effectiveness research is needed? A framework for using guidelines and systematic reviews to identify evidence gaps and research priorities. Ann Intern Med.

[3] Institute of Medicine (2011). Finding what works in health care: standards for systematic reviews.

[4] Lindsley K, Li T, Ssemanda E, Virgili G, Dickersin K (2016). Interventions for age-related macular degeneration: are practice guidelines based on systematic reviews?. Ophthalmology.

[5] Naidoo KS, Leasher J, Bourne RR, Flaxman SR, Jonas JB, Keeffe J (2016). Global vision impairment and blindness due to uncorrected refractive error, 1990-2010. Optom Vis Sci.

[6] Li T (2010). Register systematic reviews. CMAJ.

[7] American Academy of Opthalmology. Refractive errors and refractive surgery. San Francisco; 2012. https://www.aao.org/preferred-practice-pattern/refractive-errors--surgery-ppp-2013.

[8] Eden J, Levit L, Berg A, Morton S (2011). Finding what works in health care.

[9] Li T, Ervin AM, Scherer R, Jampel H, Dickersin K (2010). Setting priorities for comparative effectiveness research: a case study using primary open-angle glaucoma. Ophthalmology.

[10] Critical Appraisal Skills Programme (CASP). Critical appraisal skills programme (CASP) [internet]. 2015 [cited 10 Nov 2015]. Available from: http://www.casp-uk.net/casp-tools-checklists.

[11] Shea BJ, Grimshaw JM, Wells GA, Boers M, Andersson N, Hamel C (2007). Development of AMSTAR: a measurement tool to assess the methodological quality of systematic reviews. BMC Med Res Methodol.

[12] Moher D, Liberati A, Tetzlaff J, Altman DG, Group P (2009). Preferred reporting items for systematic reviews and meta-analyses: the PRISMA statement. Ann Intern Med.

[13] Yu T, Li T, Lee KJ, Friedman DS, Dickersin K, Puhan MA (2015). Setting priorities for comparative effectiveness research on management of primary angle closure: a survey of Asia-Pacific clinicians. J Glaucoma.

[14] Li T, Vedula SS, Hadar N, Parkin C, Lau J, Dickersin K (2015). Innovations in data collection, management, and archiving for systematic reviews. Ann Intern Med.

[15] Barsam A, Allan Bruce DS. Excimer laser refractive surgery versus phakic intraocular lenses for the correction of moderate to high myopia. Cochrane Database Syst Rev. 2014; 10.1002/14651858.CD007679.pub4. 10.1002/14651858.CD007679.pub4 PMC10726981 24937100

[16] Li S-M, Zhan S, Li S-Y, Peng X-X, Hu J, Law Hua A (2016). Laser-assisted subepithelial keratectomy (LASEK) versus photorefractive keratectomy (PRK) for correction of myopia. Cochrane Database Syst Rev.

[17] Li SM, Wu SS, Kang MT, Liu Y, Jia SM, Li SY (2014). Atropine slows myopia progression more in Asian than white children by meta-analysis. Optom Vis Sci.

[18] Shortt Alex J, Allan Bruce DS, Evans JR (2013). Laser-assisted in-situ keratomileusis (LASIK) versus photorefractive keratectomy (PRK) for myopia. Cochrane Database Syst Rev.

[19] Settas G, Settas C, Minos E, Yeung Ian YL (2012). Photorefractive keratectomy (PRK) versus laser assisted in situ keratomileusis (LASIK) for hyperopia correction. Cochrane Database Syst Rev.

[20] Walline Jeffrey J, Lindsley K, Vedula Satyanarayana S, Cotter Susan A, Mutti Donald O, Twelker JD (2011). Interventions to slow progression of myopia in children. Cochrane Database Syst Rev.

[21] Wei Mao L, Liu Jian P, Li N, Liu M. Acupuncture for slowing the progression of myopia in children and adolescents. Cochrane Database Syst Rev. 2011; 10.1002/14651858.CD007842.pub2. 10.1002/14651858.CD007842.pub2 PMC12148211 21901710

[22] Li SM, Ji YZ, Wu SS, Zhan SY, Wang B, Liu LR (2011). Multifocal versus single vision lenses intervention to slow progression of myopia in school-age children: a meta-analysis. Surv Ophthalmol.

[23] Shortt AJAB (2006). Photorefractive keratectomy (PRK) versus laser-assisted in-situ keratomileusis (LASIK) for myopia. Cochrane Database Syst Rev.

[24] Virgili G, Menchini F (2005). Laser photocoagulation for choroidal neovascularisation in pathologic myopia. Cochrane Database Syst Rev.

[25] Saw SM, Shih-Yen EC, Koh A, Tan D (2002). Interventions to retard myopia progression in children: an evidence-based update. Ophthalmology.

[26] Liu YM, Xie P (2016). The safety of orthokeratology - a systematic review. Eye and Contact Lens.

[27] Sun Y, Xu F, Zhang T, Liu M, Wang D, Chen Y, et al. Orthokeratology to control myopia progression: a meta-analysis. PLoS One. 2015;10(4):e0124535. 10.1371/journal.pone.0124535 PMC4391793 25855979

[28] Si JK, Tang K, Bi HS, Guo DD, Guo JG, Wang XR (2015). Orthokeratology for myopia control: a meta-analysis. Optom Vis Sci.

[29] Pearce MG. Clinical outcomes following the dispensing of ready-made and recycled spectacles: a systematic literature review. Clin Exp Optom. 2014;97(3):225–33. 10.1111/cxo.12126 24397254

[30] Wen D, Huang J, Li X, Savini G, Feng Y, Lin Q, Wang Q. Laser-assisted subepithelial keratectomy versus epipolis laser in situ keratomileusis for myopia: a meta-analysis of clinical outcomes. Clin Exp Ophthalmol. 2014;42(4):323–33. 10.1111/ceo.12205 24024483

[31] Chen S, Feng Y, Stojanovic A, Jankov MR, Wang Q (2012). IntraLase femtosecond laser vs mechanical microkeratomes in LASIK for myopia: a systematic review and meta-analysis. J Refract Surg.

[32] Zhang ZH, Jin HY, Suo Y, Patel SV, Montes-Mico R, Manche EE (2011). Femtosecond laser versus mechanical microkeratome laser in situ keratomileusis for myopia: Metaanalysis of randomized controlled trials. J Cataract Refract Surg.

[33] Feng YF, Chen SH, Stojanovic A, Wang QM (2012). Comparison of clinical outcomes between ‘on-flap’ and ‘off-flap’ epi-LASIK for myopia: a meta-analysis. Ophthalmologica.

[34] Feng Y, Yu J, Wang Q (2011). Meta-analysis of wavefront-guided vs. wavefront-optimized LASIK for myopia. Optom Vis Sci.

[35] Fares U, Suleman H, Al-Aqaba MA, Otri AM, Said DG, Dua HS. Efficacy, predictability, and safety of wavefront-guided refractive laser treatment: metaanalysis. J Cataract Refract Surg. 2011;37(8):1465–75. 10.1016/j.jcrs.2011.02.029 21782089

[36] Fernandes P, Gonzalez-Meijome JM, Madrid-Costa D, Ferrer-Blasco T, Jorge J, Montes-Mico R (2011). Implantable collamer posterior chamber intraocular lenses: a review of potential complications. J Refract Surg.

[37] Song YY, Wang H, Wang BS, Qi H, Rong ZX, Chen HZ. Atropine in ameliorating the progression of myopia in children with mild to moderate myopia: a meta-analysis of controlled clinical trials. J Ocul Pharmacol Ther. 2011;27(4):361–8. 10.1089/jop.2011.0017 21649523

[38] Chen SH, Feng YF, Stojanovic A, Wang QM (2011). Meta-analysis of clinical outcomes comparing surface ablation for correction of myopia with and without 0.02% Mitomycin C. J Refract Surg.

[39] Chen L, Ye T, Yang X. Evaluation of the long-term effects of photorefractive keratectomy correction for myopia in China. Eur J Ophthalmol. 2011;21(4):355–62. 10.5301/EJO.2011.6226 21240858

[40] Zhao LQ, Wei RL, Cheng JW, Li Y, Cai JP, Ma XY. Meta-analysis: clinical outcomes of laser-assisted subepithelial keratectomy and photorefractive keratectomy in myopia. Ophthalmology. 2010;117(10):1912–22. 10.1016/j.ophtha.2010.02.004 20709406

[41] Huang D, Schallhorn SC, Sugar A, Farjo AA, Majmudar PA, Trattler WB (2009). Phakic intraocular lens implantation for the correction of myopia: a report by the American Academy of ophthalmology. Ophthalmology.

[42] Solomon KD (2009). Fernandez de Castro LE, Sandoval HP, Biber JM, Groat B, Neff KD et al. LASIK world literature review: quality of life and patient satisfaction. Ophthalmology.

[43] Cui M, Chen XM, Lu P (2008). Comparison of laser epithelial keratomileusis and photorefractive keratectomy for the correction of myopia: a meta-analysis. Chin Med J.

[44] Szczotka-Flynn L, Diaz M (2007). Risk of corneal inflammatory events with silicone hydrogel and low Dk hydrogel extended contact lens wear: a meta-analysis. Optom Vis Sci.

[45] Varley GA, Huang D, Rapuano CJ, Schallhorn S, Boxer Wachler BS, Sugar A (2004). LASIK for hyperopia, hyperopic astigmatism, and mixed astigmatism: a report by the American Academy of ophthalmology. Ophthalmology.

[46] Chang MA, Jain S, Azar DT (2004). Infections following laser in situ keratomileusis: an integration of the published literature. Surv Ophthalmol.

[47] Yang XJ, Yan HT, Nakahori Y (2003). Evaluation of the effectiveness of laser in situ keratomileusis and photorefractive keratectomy for myopia: a meta-analysis. J Med Investig.

[48] Sugar A, Rapuano CJ, Culbertson WW, Huang D, Varley GA, Agapitos PJ (2002). Laser in situ keratomileusis for myopia and astigmatism: safety and efficacy: a report by the American Academy of ophthalmology. Ophthalmology.

[49] Rapuano CJ, Sugar A, Koch DD, Agapitos PJ, Culbertson WW, de Luise VP (2001). Intrastromal corneal ring segments for low myopia: a report by the American Academy of ophthalmology. Ophthalmology.

[50] Jain S, Arora I, Azar DT (1996). Success of monovision in presbyopes: review of the literature and potential applications to refractive surgery. Surv Ophthalmol.

[51] Liang GL, Wu J, Shi JT, Liu J, He FY, Xu W (2014). Implantable collamer lens versus iris-fixed phakic intraocular lens implantation to correct myopia: a meta-analysis. PLoS One.

[52] Kobashi H, Kamiya K, Hoshi K, Igarashi A, Shimizu K (2014). Wavefront-guided versus non-wavefront-guided photorefractive keratectomy for myopia: meta-analysis of randomized controlled trials. PLoS One.

[53] Zhao LQ, Zhu H, Li LM (2014). Laser-assisted subepithelial keratectomy versus laser in situ Keratomileusis in myopia: a systematic review and meta-analysis. ISRN Ophthalmol.

[54] Page MJ, Shamseer L, Altman DG, Tetzlaff J, Sampson M, Tricco AC (2016). Epidemiology and reporting characteristics of systematic reviews of biomedical research: a cross-sectional study. PLoS Med.

[55] Pussegoda K, Turner L, Garritty C, Mayhew A, Skidmore B, Stevens A (2017). Systematic review adherence to methodological or reporting quality. Syst Rev..

[56] Atkinson KM, Koenka AC, Sanchez CE, Moshontz H, Cooper H (2015). Reporting standards for literature searches and report inclusion criteria: making research syntheses more transparent and easy to replicate. Res Synth Methods.

[57] Rosman L, Twose C, Li M, Li T, Saldanha I, Dickersin K. Teaching searching in an intensive systematic review course: “how many citations should I expect to review?” (poster). Quebec City: 21st Cochrane Colloquium; 2013.

[58] Higgins JP, Altman DG, Gotzsche PC, Juni P, Moher D, Oxman AD (2011). The Cochrane Collaboration's tool for assessing risk of bias in randomised trials. BMJ.

[59] Clark HD, Wells GA, Huet C, McAlister FA, Salmi LR, Fergusson D (1999). Assessing the quality of randomized trials: reliability of the Jadad scale. Control Clin Trials.

[60] Juni P, Witschi A, Bloch R, Egger M (1999). The hazards of scoring the quality of clinical trials for meta-analysis. JAMA.

[61] Moher D, Jadad AR, Tugwell P (1996). Assessing the quality of randomized controlled trials. Current issues and future directions. Int J Technol Assess Health Care.

[62] Ioannidis JP (2016). The mass production of redundant, misleading, and conflicted systematic reviews and meta-analyses. Milbank Q.

[63] Moher D, Shamseer L, Clarke M, Ghersi D, Liberati A, Petticrew M (2015). Preferred reporting items for systematic review and meta-analysis protocols (PRISMA-P) 2015 statement. Syst Rev.

[64] Moher D, Liberati A, Tetzlaff J, Altman DG (2009). Preferred reporting items for systematic reviews and meta-analyses: the PRISMA statement. PLoS Med.

[65] Higgins JPT, Green S (editors). Cochrane Handbook for Systematic Reviews of Interventions Version 5.1.0 [updated March 2011]. The Cochrane Collaboration, 2011. Available from http://handbook.cochrane.org.

