# Supplementary material for: The quality of systematic reviews about interventions for refractive error can be improved: a review of systematic reviews
Source: BMC Ophthalmol. 2017 Sep 5;17:164. doi: 10.1186/s12886-017-0561-9 (PMC5584039; doi:10.1186/s12886-017-0561-9)
Supplement: Supplementary file 1 — Search strategies for identifying eyes and vision systematic reviews. (DOCX 42 kb) [file 12886_2017_561_MOESM1_ESM.docx]

**Online supplement 1: Search strategies for identifying eyes and vision systematic reviews**

**A. PubMed search strategy**

(ABNORMAL ACCOMMODATION[tiab] OR Abnormal color vision[tiab] OR ABNORMAL LACRIMATION[tiab] OR Abnormal vision[tiab] OR accommodative disorders[tiab] OR Amblyopia[tiab] OR Ametropia[tiab] OR ANISOCORIA[tiab] OR ANOPHTHALMIA[tiab] OR Anterior CHAMBER hemorrhage[tiab] OR Aphakia[tiab] OR aqueous outflow obstruction[tiab] OR Asthenopia[tiab] OR Balint's syndrome[tiab] OR Bilateral visual field constriction[tiab] OR Binocular Vision Disorder[tiab] OR BLEPHARITIS[tiab] OR BLEPHAROSPASM[tiab] OR BLINDNESS[tiab] OR blurred vision[tiab] OR CATARACT[tiab] OR Cataracts[tiab] OR Chorioretinal disorder[tiab] OR Chorioretinitis[tiab] OR Choroid Diseases[tiab] OR Choroidal[tiab] OR Choroiditis[tiab] OR CHROMATOPSIA[tiab] OR Color Blindness[tiab] OR Color Vision Defects[tiab] OR Color vision deficiency[tiab] OR Colour blindness[tiab] OR Conjunctival Diseases[tiab] OR CONJUNCTIVAL HAEMORRHAGE[tiab] OR Conjunctival Injury[tiab] OR CONJUNCTIVAL ULCERATION[tiab] OR CONJUNCTIVITIS[tiab] OR CORNEAL DEPOSITS[tiab] OR Corneal Diseases[tiab] OR Corneal Disorder[tiab] OR Corneal injuries[tiab] OR Corneal Injury[tiab] OR CORNEAL OEDEMA[tiab] OR CORNEAL OPACITY[tiab] OR CORNEAL ULCERATION[tiab] OR decreased Lacrimation[tiab] OR Decreased vision[tiab] OR defective vision[tiab] OR Delayed visual maturation[tiab] OR Difficulty seeing[tiab] OR difficulty with vision[tiab] OR Dim vision[tiab] OR Diminished Vision[tiab] OR DIPLOPIA[tiab] OR disturbed vision[tiab] OR ENDOPHTHALMITIS[tiab] OR EPIPHORA[tiab] OR Episcleritis[tiab] OR Equatorial staphyloma[tiab] OR EXOPHTHALMOS[tiab] OR Eye Abnormalities[tiab] OR EYE ABNORMALITY[tiab] OR Eye Burns[tiab] OR Eye disease[tiab] OR Eye Diseases[tiab] OR Eye disorder[tiab] OR Eye disorders[tiab] OR Eye edema[tiab] OR Eye Foreign Bodies[tiab] OR Eye Hemorrhage[tiab] OR EYE INFECTION[tiab] OR Eye Infections[tiab] OR Eye Injuries[tiab] OR EYE MALFORMATION[tiab] OR Eye Manifestations[tiab] OR EYE MUSCLE PARALYSIS[tiab] OR Eye Neoplasm[tiab] OR Eye Neoplasms[tiab] OR EYE PAIN[tiab] OR Eye swelling[tiab] OR Eyelid Disease[tiab] OR Eyelid Diseases[tiab] OR Eyelid Disorder[tiab] OR Eyelid Disorders[tiab] OR Eyelid pain[tiab] OR EYELID RETRACTION[tiab] OR FIXED PUPILS[tiab] OR GLAUCOMA[tiab] OR Glaucomas[tiab] OR Hazy vision[tiab] OR HEMIANOPIA[tiab] OR Hemianopsia[tiab] OR Hepatolenticular Degeneration[tiab] OR Horner's syndrome[tiab] OR HYPOPYON[tiab] OR Impaired vision[tiab] OR impaired visual acuity[tiab] OR Interference with vision[tiab] OR IRITIS[tiab] OR KERATITIS[tiab] OR KERATOCONJUNCTIVITIS[tiab] OR Keratoconus[tiab] OR Lacrimal Apparatus Diseases[tiab] OR lacrimal disorder[tiab] OR LACRIMAL DUCT OBSTRUCTION[tiab] OR legally blind[tiab] OR Lens Diseases[tiab] OR Lens Disorder[tiab] OR Lens Opacities[tiab] OR Lens Subluxation[tiab] OR Localized anterior staphyloma[tiab] OR Low vision[tiab] OR MACULAR DEGENERATION[tiab] OR MEIBOMIANITIS[tiab] OR Metastases to eye[tiab] OR MIOSIS[tiab] OR MYDRIASIS[tiab] OR MYOPIA[tiab] OR Night Blindness[tiab] OR Nystagmus[tiab] OR Ocular degeneration[tiab] OR Ocular discomfort[tiab] OR Ocular disease[tiab] OR OCULAR HAEMORRHAGE[tiab] OR OCULAR HEMORRHAGE[tiab] OR OCULAR HERPES[tiab] OR Ocular Hypertension[tiab] OR Ocular Hypotension[tiab] OR Ocular infections[tiab] OR Ocular inflammations[tiab] OR Ocular injuries[tiab] OR Ocular Injury[tiab] OR Ocular Motility Disorders[tiab] OR Ocular neoplasms[tiab] OR oculopathy[tiab] OR Open wound of ocular adnexa[tiab] OR OPHTHALMIC DISORDERS[tiab] OR Ophthalmological disorder[tiab] OR Ophthalmopathy[tiab] OR OPTIC ATROPHY[tiab] OR Optic Nerve Diseases[tiab] OR optic nerve disorder[tiab] OR Optic nerve injury[tiab] OR OPTIC NEURITIS[tiab] OR Orbital Diseases[tiab] OR PAPILLOEDEMA[tiab] OR Partial sight[tiab] OR Partial vision loss[tiab] OR Partially Sighted[tiab] OR Penetrating Eye Injuries[tiab] OR Periorbital fat herniation[tiab] OR Photalgia[tiab] OR PHOTOPHOBIA[tiab] OR PHOTOPSIA[tiab] OR PIGMENT PRECIPITATION[tiab] OR Poor vision[tiab] OR Posterior dislocation of lens[tiab] OR Posterior synechiae[tiab] OR Problem seeing[tiab] OR Proliferative Vitreoretinopathy[tiab] OR Pupil Disorders[tiab] OR Redness[All Fields] OR discharge of eye[tiab] OR Reduced ability to see[tiab] OR Reduced Vision[tiab] OR Refraction Errors[tiab] OR Refractive disorders[tiab] OR Refractive Errors[tiab] OR Retinal defects[tiab] OR RETINAL DEPOSITS[tiab] OR RETINAL DETACHMENT[tiab] OR Retinal detachments[tiab] OR Retinal Disease[tiab] OR Retinal Diseases[tiab] OR RETINAL DISORDER[tiab] OR RETINAL EDEMA[tiab] OR RETINAL HAEMORRHAGE[tiab] OR RETINAL HEMORRHAGE[tiab] OR RETINAL OEDEMA[tiab] OR RETINITIS[tiab] OR RETINOBLASTOMA[tiab] OR retinopathy[tiab] OR RETROBULBAR NEURITIS[tiab] OR Scleral Diseases[tiab] OR Scleral staphyloma[tiab] OR SCLERITIS[tiab] OR SCOTOMA[tiab] OR Sight impaired[tiab] OR Staphyloma posticum[tiab] OR STRABISMUS[tiab] OR Subnormal Vision[tiab] OR SUNKEN EYES[tiab] OR SYMBLEPHARON[tiab] OR THROMBOSIS RETINAL VEIN[tiab] OR Tunnel Vision[tiab] OR Uveal Diseases[tiab] OR Uveal Disorder[tiab] OR UVEITIS[tiab] OR vision defects[tiab] OR vision disorder[tiab] OR Vision Disorders[tiab] OR vision disturbance[tiab] OR vision impaired[tiab] OR VISION IMPAIRMENT[tiab] OR Vision problem[tiab] OR Visual Agnosia[tiab] OR visual defect[tiab] OR Visual difficulty[tiab] OR Visual disorders[tiab] OR Visual disturbance[tiab] OR VISUAL FIELD DEFECT[tiab] OR Visual Field Disorder[tiab] OR Visual field disorders[tiab] OR Visual impairment[tiab] OR Visual loss[tiab] OR Visual Pathway Disorder[tiab] OR Visual pathway disorders[tiab] OR Visual system disorder[tiab] OR VITREOUS DETACHMENT[tiab] OR VITREOUS HAEMORRHAGE[tiab] OR Vitreous Hemorrhage[tiab] OR Vitreous membranes and strands[tiab] OR Vitreous prolapse[tiab] OR Vitreous syneresis[tiab] OR Wavefront aberration[tiab] OR Weak vision[tiab] OR Wilson's Disease[tiab] OR XEROPHTHALMIA[tiab]

OR

((accommodation[tiab] OR aqueous humor[tiab] OR binocular movement[tiab] OR Capsule[tiab] OR choroid[tiab] OR ciliary body[tiab] OR conjunctiva[tiab] OR Cornea[tiab] OR (eye[tiab] AND adnexa[tiab]) OR eye[tiab] OR eyelid[tiab] OR globe[tiab] OR intraocular pressure[tiab] OR Iris[tiab] OR lacrimal passage[tiab] OR lacrimal system[tiab] OR lens[tiab] OR ocular adnexa[tiab] OR optic nerve[tiab] OR orbit[tiab] OR refraction[tiab] OR retina[tiab] OR sclera[tiab] OR trochlear nerve[tiab] OR uvea[tiab] OR Uveal Tract[tiab] OR vision[tiab] OR visual pathway[tiab] OR vitreous body[tiab] OR vitreous humor[tiab])

AND

("disease"[MeSH Terms] OR Disease[Text Word]) OR (("disease"[TIAB] NOT Medline[SB]) OR "disease"[MeSH Terms] OR Diseases[Text Word]) OR (("disease"[TIAB] NOT Medline[SB]) OR "disease"[MeSH Terms] OR Disorder[Text Word]) OR (("disease"[TIAB] NOT Medline[SB]) OR "disease"[MeSH Terms] OR Disorders[Text Word]) OR (("contusions"[TIAB] NOT Medline[SB]) OR "contusions"[MeSH Terms] OR Contusion[Text Word]) OR ("contusions"[MeSH Terms] OR Contusions[Text Word]) OR ("syndrome"[MeSH Terms] OR Syndrome[Text Word]) OR (("syndrome"[TIAB] NOT Medline[SB]) OR "syndrome"[MeSH Terms] OR Syndromes[Text Word]) OR ("dislocations"[MeSH Terms] OR Dislocations[Text Word]) OR (("dislocations"[TIAB] NOT Medline[SB]) OR "dislocations"[MeSH Terms] OR Dislocation[Text Word]) OR ((("blood vessels"[TIAB] NOT Medline[SB]) OR "blood vessels"[MeSH Terms] OR Vascular[Text Word]) AND Occlusion[All Fields]) OR ((("blood vessels"[TIAB] NOT Medline[SB]) OR "blood vessels"[MeSH Terms] OR Vascular[Text Word]) AND Occlusions[All Fields]) OR (("wounds and injuries"[TIAB] NOT Medline[SB]) OR "wounds and injuries"[MeSH Terms] OR Injury[Text Word]) OR ("injuries"[Subheading] OR ("wounds and injuries"[TIAB] NOT Medline[SB]) OR "wounds and injuries"[MeSH Terms] OR Injuries[Text Word]) OR ("coloboma"[MeSH Terms] OR Coloboma[Text Word]))

OR

"amaurosis fugax"[MeSH Terms] OR "amblyopia"[MeSH Terms] OR "asthenopia"[MeSH Terms] OR "blindness"[MeSH Terms] OR "blindness, cortical"[MeSH Terms] OR "color vision defects"[MeSH Terms] OR "conjunctival diseases"[MeSH Terms] OR "corneal diseases"[MeSH Terms] OR "diplopia"[MeSH Terms] OR "eye abnormalities"[MeSH Terms] OR "eye burns"[MeSH Terms] OR "eye diseases"[MeSH Terms] OR "eye diseases, hereditary"[MeSH Terms] OR "eye foreign bodies"[MeSH Terms] OR "eye hemorrhage"[MeSH Terms] OR "eye infections"[MeSH Terms] OR "eye injuries"[MeSH Terms] OR "eye injuries, penetrating"[MeSH Terms] OR "eye manifestations"[MeSH Terms] OR "eye neoplasms"[MeSH Terms] OR "eyelid diseases"[MeSH Terms] OR "hemianopsia"[MeSH Terms] OR "lacrimal apparatus diseases"[MeSH Terms] OR "lens diseases"[MeSH Terms] OR "night blindness"[MeSH Terms] OR "ocular hypertension"[MeSH Terms] OR "ocular hypotension"[MeSH Terms] OR "ocular motility disorders"[MeSH Terms] OR "optic nerve diseases"[MeSH Terms] OR "orbital diseases"[MeSH Terms] OR "photophobia"[MeSH Terms] OR "pupil disorders"[MeSH Terms] OR "refractive errors"[MeSH Terms] OR "retinal diseases"[MeSH Terms] OR "scleral diseases"[MeSH Terms] OR "scotoma"[MeSH Terms] OR "uveal diseases"[MeSH Terms] OR "vision disorders"[MeSH Terms] OR "vision disorders"[MeSH Terms] OR "vitreoretinopathy, proliferative"[MeSH Terms] OR "vitreous detachment"[MeSH Terms])

AND

("therapy"[Subheading] OR ("therapeutics"[TIAB] NOT Medline[SB]) OR "therapeutics"[MeSH Terms] OR treatment[Text Word]) OR (("therapeutics"[TIAB] NOT Medline[SB]) OR "therapeutics"[MeSH Terms] OR treatments[Text Word]) OR ("diagnosis"[Subheading] OR "diagnosis"[MeSH Terms] OR diagnosis[Text Word]) OR intervention[All Fields] OR interventions[All Fields] OR ("prevention and control"[Subheading] OR prevention[Text Word])

AND

(Humans[MeSH] OR (Humans[Mesh] NOT Animals[Mesh])

AND

cochrane database syst rev[ta] OR search[tiab] OR meta-analysis[pt] OR MEDLINE[tiab] OR (systematic[tiab] AND review[tiab])

**B.** **EMBASE search strategy**

((*'asthenopia'*/de OR *'asthenopia'*) OR (*'cerebral blindness'*/de OR *'cerebral blindness'*) OR (*'diplopia'*/de OR *'diplopia'*) OR (*'eye malformation'*/de OR *'eye malformation'*) OR (*'eye burn'*/de OR *'eye burn'*) OR (*'eye injury'*/de OR *'eye injury'*) OR (*'perforating eye injury'*/de OR *'perforating eye injury'*) OR (*'hemianopia'*/de OR *'hemianopia'*) OR (*'lacrimal gland disease'*/de OR *'lacrimal gland disease'*) OR (*'intraocular hypotension'*/de OR *'intraocular hypotension'*) OR (*'photophobia'*/de OR *'photophobia'*) OR (*'pupil disease'*/de OR *'pupil disease'*) OR (*'sclera disease'*/de OR *'sclera disease'*) OR (*'scotoma'*/de OR *'scotoma'*) OR (*'vitreoretinopathy'*/de OR *'vitreoretinopathy'*) OR (*'vitreous body detachment'*/de OR *'vitreous body detachment'*) OR (*'transitional blindness'*/de OR *'transitional blindness'*) OR (*'amblyopia'*/de OR *'amblyopia'*) OR (*'blindness'*/de OR *'blindness'*) OR (*'color vision defect'*/de OR *'color vision defect'*) OR (*'conjunctiva disease'*/de OR *'conjunctiva disease'*) OR (*'cornea disease'*/de OR *'cornea disease'*) OR (*'eye disease'*/de OR *'eye disease'*) OR (*'intraocular foreign body'*/de OR *'intraocular foreign body'*) OR (*'intraocular hemorrhage'*/de OR *'intraocular hemorrhage'*) OR (*'eye infection'*/de OR *'eye infection'*) OR (*'eye tumor'*/de OR *'eye tumor'*) OR (*'eyelid disease'*/de OR *'eyelid disease'*) OR (*'lens disease'*/de OR *'lens disease'*) OR (*'night blindness'*/de OR *'night blindness'*) OR (*'intraocular hypertension'*/de OR *'intraocular hypertension'*) OR (*'eye movement disorder'*/de OR *'eye movement disorder'*) OR (*'optic nerve disease'*/de OR *'optic nerve disease'*) OR (*'orbit disease'*/de OR *'orbit disease'*) OR (*'refraction error'*/de OR *'refraction error'*) OR (*'retina disease'*/de OR *'retina disease'*) OR (*'uvea disease'*/de OR *'uvea disease'*) OR (*'visual disorder'*/de OR *'visual disorder'*)) AND ((*search*) OR (*'meta analysis'* OR *'systematic review'*))
