# Supplementary material for: The quality of systematic reviews about interventions for refractive error can be improved: a review of systematic reviews
Source: BMC Ophthalmol. 2017 Sep 5;17:164. doi: 10.1186/s12886-017-0561-9 (PMC5584039; doi:10.1186/s12886-017-0561-9)
Supplement: Supplementary file 2 — Data extraction form. (PDF 676 kb) [file 12886_2017_561_MOESM2_ESM.pdf]

Project: Assessing the methodological rigor of systematic reviews on management for refractive error

Private Report Incomplete

Editing Extraction Form: Quality of systematic reviews on interventions for refractive error -- (Question 1)

Title and KQs | Publications | **Design** | Arms | Arm Details | Baseline Data | Outcomes | Outcome Details | Adverse Events | Quality | Finalize

Create Design Details Form

Please Note: This page allows you to generate extraction form questions for **Design Details**, each with an optional set of suggested responses.

Responses can be designated as drop down lists, radio buttons or check boxes, or text fields.

Display Options
Would you like to include the **Design Details** section in your extraction form? Yes

User Instructions
You may add specific instructions for data extractors using the link below.
Add/Edit Instructions

1 . **Q1. What is the objective of the systematic review?**
Record verbatim the objective of the systematic review described in one of the following sections of the article using this order of priority: Abstract; Methods/Background; Other. If not reported, insert NR in the text box.

Copy Question | Edit Question | Delete Question

2 . **Q2. What is the main conclusion(s) of the systematic review?**
Record verbatim the conclusion(s) of the systematic review described in one of the following sections of the article using this order of priority: Abstract; Discussion; Other. If not reported, insert NR in the text box.

Copy Question | Edit Question | Delete Question

3 . **Q3. In your judgment, did the authors ask at least one clearly-focused research question?**
Check "Yes" if at least the population and test intervention are specified in the review
- Yes
- No
- Can't tell

Copy Question | Edit Question | Delete Question

4 . **Q4a. Did the author(s) report having eligibility criteria for including/excluding primary studies in the systematic review?**
Answer 'Yes' for all Cochrane reviews. If "Yes" is selected, answer Q4b. IF "NO" OR "CAN'T TELL" ARE SELECTED, SKIP TO Q5a.
- Yes
- No
- Can't tell

Copy Question | Edit Question | Delete Question

5 . **Q4b. Did the author(s) say that the eligibility criteria were pre-specified?**
Answer 'Yes' for all Cochrane reviews. If a protocol or design paper with eligibility criteria is cited, check 'Yes'.
- Yes
- No
- Can't tell

Copy Question | Edit Question | Delete Question

6 . **Q5a. Did the authors report that they planned to include participants of the following ages?**

This question asks about the eligibility criteria rather than the results. Please only choose what is EXPLICITLY stated.

- All participants <12 years
- All participants ≥12 years
- Participants both younger and older than 12 years
- Can't tell

Copy Question | Edit Question | Delete Question

7 . **Q5b. Did the authors report that they planned to include participants with any of the following CONDITIONS?**

This question asks about the eligibility criteria rather than the results. Check "Yes", "No," or "Can't tell" for each category. Please only choose what is EXPLICITLY stated.

	Yes	No	Can't tell
5b1. Astigmatism	○	○	○
5b2. Hyperopia	○	○	○
5b3. Myopia	○	○	○
5b4. Presbyopia	○	○	○

Copy Question | Edit Question | Delete Question

8 . **Q6. Did the authors report that they planned to examine any of the following interventions?**

Select "Yes", "No," or "Can't tell" for each category. Please only choose what is EXPLICITLY stated. If yes, specify the exact type of intervention (e.g., LASIK) and specify if multiple types of the intervention were compared (e.g., soft versus rigid contact lenses).

	Types of interventions	If yes, specify
Q6a. Acupuncture	Select...	
Q6b. Contact lenses	Select...	
Q6c Eyeglasses	Select...	
Q6d. Patching	Select...	
Q6e. Pharmaceuticals	Select...	
Q6f. Surgery	Select...	
Other (please specify):		

Copy Question | Edit Question | Delete Question

9 . **Q7. Did the authors report that they planned to examine any of the following outcomes?**

Look in the METHODS rather than the results section.

	Yes	No	Can't tell
Q7a. Visual acuity: Uncorrected visual acuity (UCVA)	○	○	○
Q7b. Visual acuity: Best corrected visual acuity (BCVA)	○	○	○
Q7c. Visual acuity: Usual (habitual) corrected visual acuity	○	○	○
Q7d. Visual acuity: Mean postoperative spherical equivalent	○	○	○
Q7e. Visual acuity: Patient acceptance of correction	○	○	○
Q7f. Refractive error (e.g., achieving target refraction, proportion of eyes within ±0.50 D of target refraction)	○	○	○
Q7g. Need for correction (e.g., proportion of	○	○	○

patients continue to wear glasses or contact lenses)			
Q7h. Contrast sensitivity	○	○	○
Q7i. Patient satisfaction (appearance, comfort)	○	○	○
Q7j. Quality of life / vision-related quality of life	○	○	○
Q7k. Functioning (e.g., reading, driving, mobility, activities of daily living)	○	○	○
Q7l. Adverse events (e.g., corneal ectasia, discomfort, infection, induced astigmatism, subepithelial haze, IOP, dry eye, pain, etc.)	○	○	○
Q7m. Cost effectiveness of intervention	○	○	○
Other (please specify):			

Copy Question | Edit Question | Delete Question

10 . **Q8. Did the authors report that they planned to include the following types of study design to answer the main research question(s) as defined in the objective?**

Please see question 1 for defined objective

	Yes	No	Unclear
Q8a. Randomized controlled trial (RCT)	○	○	○
Q8b. Quasi-randomized controlled trial	○	○	○
Q8c. Observational study (including cohort study, case-control study, cross-sectional study, information from patient registries, case-series, and patient testimonials)	○	○	○
Other (please specify):			

Copy Question | Edit Question | Delete Question

11 . **Q9a. Did the authors report that they searched any of the following bibliographic databases?**

	Yes	No	Unclear
Q9a1. PubMed or MEDLINE	○	○	○
Q9a2. The Cochrane Library or the Cochrane Central Register of Controlled Trials (CENTRAL)	○	○	○
Q9a3. EMBASE	○	○	○

Q9a4. LILACS	○	○	○
Q9a5. Any other bibliographic database(s)	○	○	○

Copy Question | Edit Question | Delete Question

12 . **Q9b. What was the total number of bibliographic databases searched?**

Please enter the number of databases explicitly stated in the methods or enter "not reported". BIBLIOGRAPHIC DATABASE is defined as a database which provides descriptive records of items such as book(chapter)s, articles and conference proceedings. Do NOT count clinical trial registers, Science Citation Index, or reference lists as bibliographic databases.

-- Make Your Selection --

Copy Question | Edit Question | Delete Question

13 . **Q9c. What was the latest date of searching any bibliographic databases?**

Please select one option from each drop down menu. If the month, date, or year is not reported, select "not reported" for that item.

	Month	Date	Year
Date	Select...	Select...	Select...

Copy Question | Edit Question | Delete Question

14 . **9d-9i. Did the authors report using any of the following methods to identify relevant studies?**

	Yes	No	Unclear
9d. Searched for non-English-language studies for at least one of the above bibliographic database(s)	○	○	○
9e. Searched for all relevant years (i.e., all years after which the interventions became available) for at least one of the above bibliographic database	○	○	○
9f. Searched reference lists, or searched for reports that cited included studies	○	○	○
9g. Contacted experts in the field and/or contacted study authors	○	○	○
9h. Searched for unpublished or difficult-to-access studies (e.g., grey literature, FDA data, internal company reports, conference abstracts)	○	○	○
9i. Searched for ongoing studies (e.g., clinicaltrials.gov, WHO search portal)	○	○	○

Other (please specify):

Copy Question | Edit Question | Delete Question

15 . **9. Overall, in your judgment, do you think the search for evidence was reasonably comprehensive?**

To answer this question, you are encouraged to consider additional information (e.g., search terms, number of trials identified).

- ○ Yes
- ○ No
- ○ Can't tell

Copy Question | Edit Question | Delete Question

16 . **10. Did the authors report that they planned to assess the risk of bias in individual studies?**

Many methods exist to assess the risk of bias, including scales, in which various components of quality are scored and combined to give a summary score; or checklists, in which specific questions are asked; or domain-based evaluation, in which critical assessments are made separately for different domain, for example, allocation concealment, masking.

- Yes
- No
- Can't tell

Copy Question | Edit Question | Delete Question

17 . **11a1. Did the authors report how many reviewers were involved in assessing each title and/or abstract's eligibility?**

If "2 or more" is selected, answer question 11a2

- 1
- 2 or more
- Not reported

Copy Question | Edit Question | Delete Question

18 . **11a2. Did the authors report that reviewers worked independently in assessing each title and/or abstract's eligibility?**

- Yes
- No
- Can't tell

Copy Question | Edit Question | Delete Question

19 . **11b1. Did the authors report how many reviewers were involved in assessing each full text article's eligibility?**

If "2 or more" is selected, answer question 11b2

- 1
- 2 or more
- Not reported

Copy Question | Edit Question | Delete Question

20 . **11b2. Did the authors report that reviewers worked independently in assessing each full text article's eligibility?**

- Yes
- No
- Can't tell

Copy Question | Edit Question | Delete Question

21 . **11c1. Did the authors report how many reviewers were involved in assessing the risk of bias of each included study?**

If "2 or more" is selected, answer question11c2

- 1
- 2 or more
- Not reported

Copy Question | Edit Question | Delete Question

22 . **11c2. Did the authors report that reviewers worked independently in assessing the risk of bias of each included study?**

- Yes
- No
- Not applicable, risk of bias was not assessed
- Can't tell

Copy Question | Edit Question | Delete Question

23 . **11d1. Did the authors report how many reviewers were involved in abstracting data from each included study?**

If "2 or more" is selected, answer question 11d2

- 1
- 2 or more
- Not reported

Copy Question | Edit Question | Delete Question

24 . **11d2. Did the authors report that reviewers worked independently in abstracting data from each included study?**

- Yes
- No
- Can't tell

Copy Question | Edit Question | Delete Question

25 . **12. How many primary studies were included in the systematic review?**

Enter "Can't tell" if the reporting was unclear and you cannot figure out the number of primary studies included.

-- Make Your Selection --

Copy Question | Edit Question | Delete Question

26 . **13a. How many PARTICIPANTS were included in the systematic review?**

Enter "Can't tell" if the reporting was unclear and you cannot figure out how many participants were included.

Copy Question | Edit Question | Delete Question

27 . **13b. How many EYES were included in the systematic review?**

Enter "Can't tell" if the reporting was unclear and you cannot figure out how many eyes were included.

Copy Question | Edit Question | Delete Question

28 . **14. Did the authors describe/present the characteristics of the included studies?**

Examples of study characteristics include patient age, race, sex, relevant socioeconomic data; disease status, duration, severity; interventions, outcomes, and risk of bias.

- Yes
- No
- Can't tell

Copy Question | Edit Question | Delete Question

29 . **15. What comparisons were made in the systematic review? Please specify**

Look in the RESULTS rather than the methods section. Extract the results verbatim (e.g., copy and paste).

Copy Question | Edit Question | Delete Question

30 . **16a-16b. How did the authors combine the results?**

If "Yes" is selected for 16b, answer 16c-16e.

	Yes	No	Can't tell	Not applicable
16a. Qualitatively (the authors described characteristics and risk of bias in individual studies that may affect the cumulative evidence)	○	○	○	○
16b. Quantitatively (meta-analysis)	○	○	○	○

Copy Question | Edit Question | Delete Question

31 . **16c-16e. How did the authors combine the results?**

	Yes	No	Can't tell
16c. Reported statistical heterogeneity?	○	○	○
16d. Given the clinical and statistical heterogeneity, was it reasonable to combine results in a meta-analysis?	○	○	○
16e. Were the methods used to combine results of studies appropriate? (Select "Yes" if correct variance and meta-analysis formula were used, and treatment effects were meta-analyzed at trial level (instead of combining results from the same arm))	○	○	○

Copy Question | Edit Question | Delete Question

32 . **17. Did the authors report doing a sensitivity analysis?**

- Yes
- No
- Can't tell

Copy Question | Edit Question | Delete Question

33 . **18. Did the authors report doing a subgroup analysis?**

- Yes
- No
- Can't tell

Copy Question | Edit Question | Delete Question

34 . **19. Did the authors report doing a meta-regression?**

- Yes
- No
- Can't tell

Copy Question | Edit Question | Delete Question

35 . **20. Did the authors discuss the limitations of the systematic review at the study and outcome level?**

E.g., discuss risk of bias in individual studies and heterogeneity across studies

- Yes
- No
- Can't tell

Copy Question | Edit Question | Delete Question

36 . **21. Did the authors discuss the limitations of the systematic review at the review level?**

E.g., discuss incomplete retrieval of relevant studies, publication bias etc.

- Yes
- No
- Can't tell

Copy Question | Edit Question | Delete Question

37 . **22a. Did the authors report the source(s) of monetary or material support for the systematic review?**

if "Yes" is selected, answer question 22b.

- Yes
- No

Copy Question | Edit Question | Delete Question

38 . **22b. What was the reported source(s) of monetary or material support for the systematic review?**

	Source(s) of monetary or material support for the systematic review
22b1. Government (e.g., National Institutes of Health)	Select...
22b2. Pharmaceutical industry	Select...
22b3. Other industry	Select...
22b4. Foundation	Select...
22b5. Academic department or institution	Select...
22b6. No funding	Select...
22b7. Not reported	Select...
22b8. Other, specify	

Copy Question | Edit Question | Delete Question

39 . **23a. Did the authors report explicitly that none of the authors has any financial interests?**

- Yes
- No

Copy Question | Edit Question | Delete Question

40 . **23b. Did the authors report any type(s) of financial interests?**

If "Yes" is selected, answer questions 23c-23p

- Yes
- No

Copy Question | Edit Question | Delete Question

41 . **23c-23p: If 23b is yes, please specify type of financial interests.**

	Specify type of interest
23c. Board membership	Select...
23d. Consultancy	Select...

23e. Employment — Select...

23f. Expert testimony — Select...

23g. Gifts — Select...

23h. Grants/grants pending — Select...

23i. Honoraria — Select...

23j. Payment for manuscript preparation — Select...

23k. Patents (planned, pending or issued) — Select...

23l. Royalties — Select...

23m. Payment for development of educational presentations including service on speakers' bureaus — Select...

23n. Stock/stock options — Select...

23o. Travel/accommodations expenses covered or reimbursed — Select...

23p. Other, specify

Copy Question | Edit Question | Delete Question

42. **24. In your judgment, do you think the conclusions related to main research question of the systematic review are supported by the data?**

- Yes
- No
- Can't tell

Copy Question | Edit Question | Delete Question

43. **25a. Overall, in your judgment, do you think the systematic review is reliable?**

If "No" is selected, answer 25b to 25g.

- Yes
- No, because...

Copy Question | Edit Question | Delete Question

44. **25b-25g. Why do you think the systematic review is not reliable?**

	The systematic review is not reliable because
25b. Did not define eligibility criteria (see question 4)	Select...
25c. Did not conduct a comprehensive search (see question 9j)	Select...
25d. Did not assess risk of bias of included studies (see question 10)	Select...
25e. Used inappropriate quantitative methods to combine findings of included studies (see question 16)	Select...
25f. Sources of monetary or material support for the systematic review and/or author(s) placed the review at high risk of bias (see questions 22&23)	Select...
25g. Other, specify	

Copy Question | Edit Question | Delete Question

45. **26. Extractor's name:**

Copy Question | Edit Question | Delete Question

46. **27. Date form completed**

mm/dd/yyyy

Copy Question | Edit Question | Delete Question

Add a New Design Detail

AHRQ

Accessibility Disclaimers EEO FOIA Inspector General
HHS Digital Strategy Plain Writing Act Privacy Policy
Electronic Policies Viewers & Players

Get Social

About Us
Careers
Contact Us
Sitemap
FAQ

Health Care Information
For Patients & Consumers
For Professionals
For Policymakers

Research Tools & Data
Funding & Grants
Offices, Centers & Programs
News & Events

U.S. Department of Health & Human Services
The White House
USA.gov: The U.S. Government's Official Web Portal

Agency for Healthcare Research and Quality
5600 Fishers Lane
Rockville, MD 20857
Telephone: (301) 427-1364
